# Hospitalizations in Pediatric and Adult Patients for All Cancer Type in Italy: The EPIKIT Study under the E.U. COHEIRS Project on Environment and Health †^,^‡

**DOI:** 10.3390/ijerph14050495

**Published:** 2017-05-09

**Authors:** Prisco Piscitelli, Immacolata Marino, Andrea Falco, Matteo Rivezzi, Roberto Romano, Restituta Mazzella, Cosimo Neglia, Giulia Della Rosa, Giuseppe Pellerano, Giuseppe Militerno, Adriana Bonifacino, Gaetano Rivezzi, Roberto Romizi, Giuseppe Miserotti, Maurizio Montella, Fabrizio Bianchi, Alessandra Marinelli, Antonella De Donno, Giovanni De Filippis, Giuseppe Serravezza, Gianluca Di Tanna, Dennis Black, Valerio Gennaro, Mario Ascolese, Alessandro Distante, Ernesto Burgio, Massimo Crespi, Annamaria Colao

**Affiliations:** 1 Medicina Futura Research, Southern Italy Hospital Institute (IOS), Centro Direzionale, Isola E3, Palazzo Avalon, 80143 Naples, Italy; falco.and@gmail.com (A.F.); matteo.rivezzi@gmail.com (M.R.); 2 Department of Economics and Statistics and CSEF, University Federico II, 80131 Naples, Italy; miss.immamarino@gmail.com; 3 Euro Mediterranean Scientific Bio-Medical Institute, ISBEM, 72023 Mesagne (Brindisi), Italy; wilksbartlett@gmail.com (R.R.); resty1988@libero.it (R.M.); neglia@isbem.it (C.N.); dellarosa.giulia@gmail.com (G.D.R.); giuspelle@libero.it (G.P.); distante@isbem.it (A.D.); 4 Local Health Authority ASL Napoli 3 South, 80100 Naples, Italy; g.militerno@gmail.com; 5 St. Andrea Hospital, La Sapienza University, 00185 Rome, Italy; adriana.bonif@gmail.com; 6 Division of Neonatology, St. Anna & St. Sebastiano Hospital, 81100 Caserta, Italy; isdecaserta@gmail.com; 7 Local Health Authority USL 8, 52100 Arezzo, Italy; isde@ats.it; 8 Local Health Authority USL Piacenza, 29121 Piacenza, Italy; giuseppe.miserotti@gmail.com; 9 IRCCS G. Pascale Foundation, National Cancer Institute, 80131 Naples, Italy; m.montella@istitutotumori.na.it; 10 National Research Council, CNR-IFC, 56121 Pisa, Italy; fabrizio.bianchi@ifc.cnr.it; 11 Department of Experimental Medicine, Second University of Naples (SUN), 80138 Naples, Italy; alessandra.marinelli@unina2.it; 12 Department of Biological and Environmental Sciences and Technologies (DISTEBA), University of Salento, 73100 Lecce, Italy; antonella.dedonno@unisalento.it; 13 Local Health Authority ASL LE, 73100 Lecce, Italy; g.defilippis@gmail.com (G.D.F.); info@legatumorilecce.org (G.S.); 14 Centre of Primary Care and Public Health, Queen Mary University of London, London E14NS, UK; g.ditanna@qmul.ac.uk; 15 Department of Epidemiology and Biostatistics, University of California San Francisco (UCSF), San Francisco, CA 94158, USA; dblack@psg.ucsf.edu; 16 National Cancer Institute IRCCS San Martino, 16121 Genova, Italy; valerio.gennaro@hsanmartino.it; 17 Division of Pediatric Surgery, Salerno University Hospital “Ruggi D’Aragona”, 84100 Salerno, Italy; ascomar@tiscali.it; 18 European Cancer and Environment Research Institute (ECERI), 21004 Bruxelles, Belgium; erburg@libero.it; 19 Collegium Ramazzini, Bologna 40010, Italy; maxcrespi35@gmail.com; 20 Department of Clinical Medicine and Surgery, University Federico II, 80131 Naples, Italy; colao@unina.it

**Keywords:** hospitalizations, cancer incidence, children, pediatric cancer, adult cancer, environment and health

## Abstract

*Background*: Cancer Registries (CRs) remain the gold standard for providing official epidemiological estimations. However, due to CRs’ partial population coverage, hospitalization records might represent a valuable tool to provide additional information on cancer occurrence and expenditures at national/regional level for research purposes. The Epidemiology of Cancer in Italy (EPIKIT) study group has been built up, within the framework of the Civic Observers for Health and Environment: Initiative of Responsibility and Sustainability (COHEIRS) project under the auspices of the Europe for Citizens Program, to assess population health indicators. *Objective*: To assess the burden of all cancers in Italian children and adults. *Methods*: We analyzed National Hospitalization Records from 2001 to 2011. Based on social security numbers (anonymously treated), we have excluded from our analyses all re-hospitalizations of the same patients (*n* = 1,878,109) over the entire 11-year period in order to minimize the overlap between prevalent and incident cancer cases. To be more conservative, only data concerning the last five years (2007–2011) have been taken into account for final analyses. The absolute number of hospitalizations and standardized hospitalization rates (SHR) were computed for each Italian province by sex and age-groups (0–19 and 20–49). *Results*: The EPIKIT database included a total of 4,113,169 first hospital admissions due to main diagnoses of all tumors. The annual average number of hospital admissions due to cancer in Italy has been computed in 2362 and 43,141 hospitalizations in pediatric patients (0–19 years old) and adults (20–49 years old), respectively. Women accounted for the majority of cancer cases in adults aged 20–49. As expected, the big city of Rome presented the highest average annual number of pediatric cancers (*n* = 392, SHR = 9.9), followed by Naples (*n* = 378; SHR = 9.9) and Milan (*n* = 212; SHR = 7.3). However, when we look at SHR, minor cities (i.e., Imperia, Isernia and others) presented values >10 per 100,000, with only 10 or 20 cases per year. Similar figures are shown also for young adults aged 20–49. *Conclusions*: In addition to SHR, the absolute number of incident cancer cases represents a crucial piece of information for planning adequate healthcare services and assessing social alarm phenomena. Our findings call for specific risk assessment programs at local level (involving CRs) to search for causal relations with environmental exposures.

## 1. Introduction

Until 1955, the word “tumor” was generically defined as “an occupational disease of chemical industry workers” in the most prestigious encyclopedic dictionaries [1]. Nowadays, cancer is generally associated with old age, and its continuous increase—observed throughout the 20th century in all industrialized countries—is generally explained as a consequence of progressive accumulation of oxidative, stochastic (random) genetic damage, along with ongoing improvement in our diagnostic capacities. From the end of the 1980s to date, cancer has involved individuals of all ages, including younger people, whose number it is difficult to estimate [2]. Cancer incidence data are essential for epidemiological purposes, as well as for planning screening campaigns and cancer primary prevention or surveillance. The implementation of Cancer Registries (CRs) represents the gold standard methodology for data collection and cancer surveillance at the local level [3]. 

In Italy, a network of population-based local cancer registries has been established (Italian Association of Cancer Registries, AIRTUM) in order to set high qualitative standards in data collection that result in reliable reports, with data available on the AIRTUM website. However, the AIRTUM CRs does not cover the entire Italian population, with a remarkable difference in CRs population coverage among Northern (50.2%), Central (25.5%) and Southern areas of the Country (17.9%) [4]. In the last decade, cancer incidence estimation at national level have been provided in the frame of a specific cooperation between the National Institute of Public Health (ISS), the National Cancer Institute of Milan, and the AIRTUM. They mainly adopted the Mortality-Incidence Analysis MODel (MIAMOD) statistical model, which represents a back-calculation approach to estimate the morbidity of chronic irreversible diseases from existing mortality and survival data [5,6]. 

Referring only to CRs when searching for epidemiological data about the overall and cancer-specific burden of tumors in general population or in well-defined subgroups (i.e., pediatric population or younger adults) might represent a limitation, due to the problem of CRs’ partial population coverage [7]. Despite possible limitations related to underestimation produced by the proportion of cancer patients that is not hospitalized, additional secondary databases such as hospital discharge records (HDR) have been proposed by researchers as potential tools to improve the ability of assessing the burden of several diseases, including cancer [8,9,10,11,12]. The accuracy of these secondary data sources has been specifically explored [13,14]. A study carried out by Penberthy et al. used both CRs and HDR for the detection of incident cancer cases [15]. In our previous studies, we have used HDR as secondary data source to specifically address the issue of breast cancer [12,16]. 

In this paper, we present the first analyses performed on the national hospital discharge records maintained at central level by the Ministry of Health concerning hospitalization due to main diagnosis of overall cancer, as a result of the work carried out by the Epidemiology of Cancer in Italy (EPIKIT) study group. This latter initiative has been promoted within the framework of the European Civic Observers for Health and Environment: Initiative of Responsibility and Sustainability (COHEIRS) project under the auspices of the European Union’s “Europe for Citizens” Program [17]. COHEIRS—coordinated by ALDA, the European Association of Local Democracy Agencies at the European Council in Strasbourg was implemented in Italy by the Euro Mediterranean Scientific Bio-Medical Institute (ISBEM) and International Society Doctors for the Environment (ISDE) and it has been acknowledged as one of the three best European projects of the year 2013. The aim of the COHEIRS project was to foster the implementation of the “precautionary principle” (with specific focus on health and environment assessment) stated in the Maastricht Treaty and at Article 191 of the European Union Treaty [17]. Precautionary principle should be invoked when scientific final proofs of toxicity for the environment or health are lacking, but some evidence lead to possible concerns.

Although CRs remain the gold standard methodology to collect epidemiological information on cancer incidence at local level, we attempted to estimate the burden of cancer at regional and province level for the entire nation thanks to the specific expertise developed by our study group in the treatment and analysis of HDR. These analyses could also be useful in better understanding the consistency of social alarm that have spread in certain areas of the country (i.e., the Campania region) concerning possible environmental threats to human health related to illegal activities leading to soil/air/water pollution. Our work could help in explaining the widespread perception of higher incidence of tumors in pediatric population (0–19 years old) and adults belonging to those age groups (20–49 years old) generally excluded by official screening for cancer prevention. At the same time, our work could be used by decision makers in planning healthcare services to be offered at local level in the field of oncology.

## 2. Materials and Methods

### 2.1. Database

Information concerning hospitalizations occurring in Italian hospitals are registered in Hospital Discharge Records (HDRs), which are collected in the Italian Ministry of Health’s national hospitalization database. The information is anonymous and includes the region and hospital where the patients have been hospitalized, type of hospitalization (ordinary admission or day hospital), region and province where the patient come from, local health authority (ASL) who is paying for the hospitalization costs, patient’s age, gender, main diagnosis, secondary diagnoses (comorbidities which are not the cause of the hospitalization), procedures performed, diagnosis related group (DRG) and length of the hospitalization. HDRs are kept at the central level by the Ministry of Health since the year 1999, but the national hospitalization database has been fully implemented for all Italian regions only since 2001. It is important to point out that, in the national hospitalization database, people admitted at hospitals located in different region or provinces (different from those ones where patients live), are classified according to their hometown address. Therefore, there was no possibility of misclassification of patients from one province in another. However, it was impossible to assess the time people have been living in a specific region or province.

The Italian Ministry of Health has officially provided ISDE Campania (who is part of the COHEIRS Project and promoted the EPIKIT study group) with the full database covering all hospitalizations occurred in Italy between 2001 and 2011 due to cancer diagnoses. The quality of these data is known to be very high and certified at the central level by the Ministry of Health, with completeness and reliability of records (in terms of correspondence between hospitalizations and individual social security numbers as well as in terms of absence of errors or missing data) varying from 95.6% (year 2001) and 99.8% (from year 2008), respectively, as reported in our previous studies [9,17].

Our dataset included all hospitalized patients identified based on the following International Classification of Diseases (ICD-9-CM) major diagnosis codes: 174 (breast cancer), 162 (lung cancer), 163 (pleural cancer), 161 (larynx cancer), 146 (oropharyngeal cancer), 147 (rhino-pharyngeal cancer), 148 (hypo-pharyngeal cancer), 141 (tongue cancer), 142 (salivary glands cancer), 193 (thyroid cancer), 01, 02, 03, 04, 05 (brain cancer), 188 (bladder cancer), 185 (prostatic cancer), 180 (uterine cervix cancer), 182 (uterine cancer), 183 (ovary cancer), 153 (colon cancer), 157 (pancreatic cancer), 186.0 and 186.9 (testicular cancer), 189 (kidney and urinary tract cancer), 155 (liver cancer), 200.0–200.2, 201.0–201.9, 202.0, 203.0–203.1, 203.8, 204.0, 204.2, 204.8, 204.9, 205.0, 205.1–205.3, 205.8, 205.9, 206.0–206.3, 206.8–206.9, 207, 208 (malignant tumors of lymphatic and hemopoietic system), 1510–1519 (gastric cancer), 1501–1509 (esophageal cancer), 1580–1589 (peritoneal cancer), 1560–1569 (gall-bladder and biliary tract cancer). We considered both ordinary hospitalization and day hospital regimens. 

Based on social security numbers (which were treated anonymously), the Ministry of Health has enabled us to exclude all hospital re-admissions of the same patient over the entire study period, in order to minimize possible bias related to the overlapping between prevalent and incident cancer cases. To exclude hospital re-admissions from our analysis, we have considered as hospitalization “index” only the first hospital admission over the entire study period (2001–2011). Patients presenting the same social security number (treated anonymously) and the same major diagnosis were considered as the same person, and they were computed only one time. This kind of approach to minimize the overlapping between prevalent and incident cases has been already used and validated by the Environmental Protection Agency of Piemonte Region for the assessment of population health indicators [18]. After having identified first hospital admissions for the cancer diagnosis that occurred between 2001 and 2011, we removed relapses and admissions for previous cancer patients from hospitalizations taking place during the entire 11-year period. To be more conservative and exclude prevalent cancer cases and disease relapses, we have included in our final analyses only the last five years (2007–2011). 

### 2.2. Analyses Performed and Data Treatment

The total number of records contained in the official database provided by the Ministry of Health were 5,991,278. About 24,194 records were missing information concerning the province where the hospitalized patient was living. We have excluded from our analyses all re-hospitalizations concerning the same patient (*n* = 1,878,109) over the entire 11-year period. As a result, the Epikit Database contains a total of 4,113,169 “first hospital admissions” due to main diagnoses of any cancer detailed in the previous paragraph. The absolute frequencies (number of hospitalizations) were computed for each Italian region (*R*) and province (*P*), by sex (*S*), year (*y*), and 10-year age groups (*x*):

(1) $$n_{y,x}^{S}\left(Reg\;or\;Prov\right)$$

The standardized hospitalization rate (*H*) per 100,000 inhabitants was computed by referring to the Italian population as standard $Pop_{y,x}^{S}\left(IT\right)$ of year 2001 (*y*) per age group (*x*) and sex (*S*):

(2) Hy,xRS=[∑xhy,xRS]*[Popy,xS(IT)][∑xPy,xS(IT)]×100

(3) Hy,xPS=[∑xhy,xPS]*[Popy,xS(IT)][∑xPy,xS(IT)]×100

Data were analyzed and processed using Stata (StataCorp, College Station, TX, USA) and Excel (Microsoft, Redmond, WA, USA) software. Age and sex standardized rates per region and province were calculated based on population data provided by the Italian National Institute for Statistics (ISTAT). The results of the analyses in this first paper have been studied as cumulative data (all tumors) per each Italian region and province according to sex and age groups (0–19; 20–49). Data are specifically presented per province (in tables and on maps) as absolute number of hospitalizations and standardized hospitalization rates for each of the years from 2007 to 2011. 

## 3. Results

**Figure 1:**
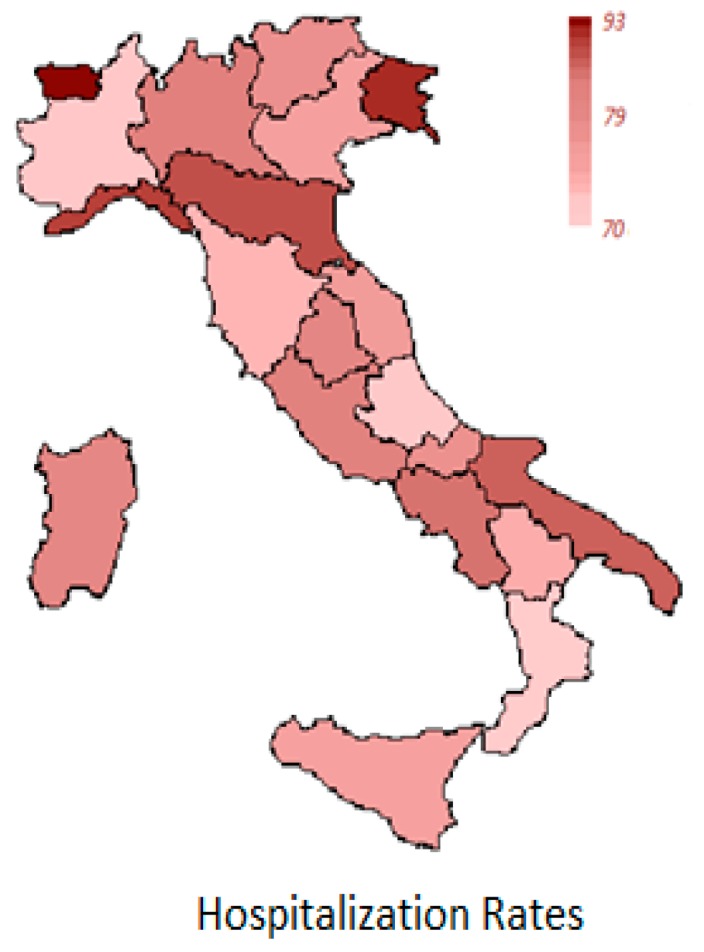
Overall regional cancer standardized hospitalization rates (SHR) per 100,000 in the population aged 0–100 years old (average annual value).

Table 1 and Figure 1 report the annual average standardized hospitalization rate (SHR) per region due to all cancers in people aged 0–100 years old. Table 2 and Table 3 present the overall number of new hospitalizations and the corresponding standardized hospitalization rates per 100,000 inhabitants per province due to all cancers in pediatric population aged 0–19 and adults aged 20–49 years old, respectively.

The average annual number of hospitalizations due to all cancers in Italy was 2362 in pediatric populations (0–19 years old) and 43,141 in adults aged 20–49 years old. Women accounted for the majority of cancer cases in young adults 20–49 (data not presented). As expected, in terms of absolute number of hospitalizations, the biggest cities of Rome, Naples, and Milan display the highest values in both the examined age groups, followed by smaller cities (number of pediatric hospitalizations > 100) such as Turin, Bari, Salerno, Caserta, Catania and Palermo. All these cities were always at the top ten places in both the examined age groups, Particularly, Rome presented the highest average annual number of pediatric cancers (*n* = 392, SHR = 9.9), followed by Naples (*n* = 378; SHR = 9.9) and Milan (*n* = 212; SHR = 7.3). Rome displayed also the highest number of average annual hospitalizations due to cancer in young adults aged 20–49 years old (*n* = 3633; SHR = 89.0), followed by Milan (*n* = 2491; SHR = 82.4), Naples (*n* = 2398; SHR = 74.8), Turin (*n* = 1376; SHR = 63.6), and minor cities such as Bari (*n* = 1103; SHR = 85.8), Catania (*n* = 924; SHR = 83.2), Brescia (*n* = 906; SHR = 71.1), Salerno (*n* = 781; SHR = 69.3), Bologna (*n* = 735; SHR = 77.5), Caserta (*n* = 673; SHR = 69.4), Lecce (*n* = 645; SHR = 81.1), Bergamo (*n* = 645; SHR = 57.5), Florence (*n* = 635; SHR = 68.8), Padova (*n* = 629; SHR = 66.9), Verona (*n* = 617; SHR = 67.2), Genova (*n* = 614; SHR = 80.0).

When looking only at SHR of pediatric cancers, minor cities (i.e. Imperia, Isernia and others) presented values >10 per 100,000, with only 20 cases per year. Despite huge differences in the number of hospitalizations, when looking at the SHR in pediatric patients, the highest values (≥8 per 100,000) were recorded in provinces belonging to well defined areas: Isernia, Naples, Caserta, Salerno, Avellino (region Molise and Campania); Rome, Frosinone, Rieti, Latina, Viterbo and Terni (Region Lazio and Umbria); Bari, Foggia, Barletta/Andria and Brindisi (Puglia); L’Aquila, Teramo, Chieti, Ascoli Piceno (Abruzzo and Marche); Parma (Emilia Romagna); Aosta (Val d’Aosta Region); Vercelli, and Imperia (Piemonte and Liguria); Cagliari, Oristano, Ogliastra, Medio Campidano (Sardinia); Catania, Messina, Trapani, Enna, Caltanissetta (Sicily).

Similarly, also when looking at the SHR in adult patients aged 20–49 years old, the highest values (≥75 per 100,000) were displayed in the entire Sardinia island, Liguria and Marche Region, and in the following Provinces: Milan, Alessandria, Udine, Gorizia, Ravenna, Ferrara, Rimini, Piacenza, Rovigo, Bologna and Parma (in Northern Italy); Rome, Frosinone, Rieti, Latina, L’Aquila, Lucca, Livorno, Massa-Carrara, Perugia and Terni (Central Italy); Messina, Enna, Caltanissetta, Catania, Campobasso, Bari and Lecce (in Southern Italy).

**Figure 2:**
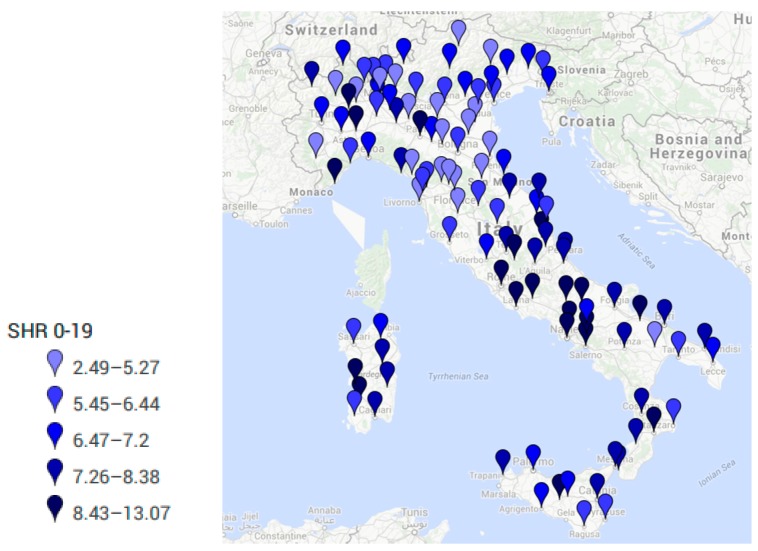
Standardized Hospitalization Rate (SHR) per 100,000 per each Italian province due to main diagnosis of cancer diagnosis of (all tumors) in pediatric population (0–19 years old).

**Figure 3:**
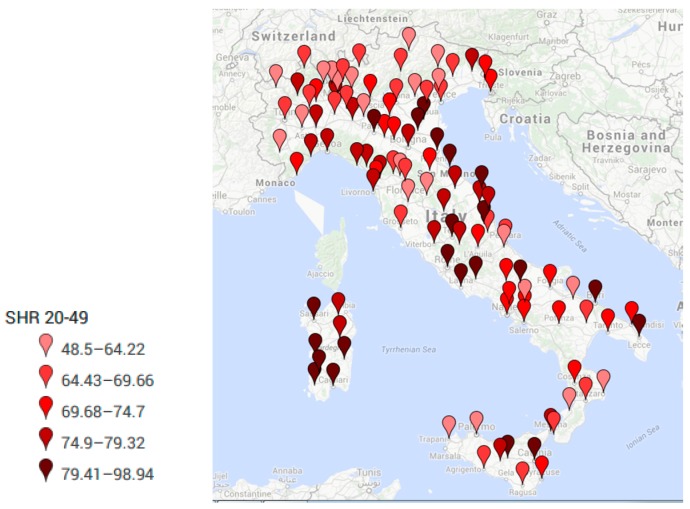
Standardized Hospitalization Rate (SHR) per 100,000 inhabitants per each Italian province due to main diagnosis of cancer (all tumors) in adults aged 20–49 years old.

Women always presented SHR values higher than men (data not presented). Figure 2 and Figure 3 show on a map the average annual value of standardized hospitalization rate (SHR) per 100,000 inhabitants per province in pediatric population aged 0–19 and in young adults 20–49 due to all cancers.

## 4. Discussion

Our objective was to provide a preliminary tabulation of hospitalizations occurred in Italy from 2007 to 2011. The specific goal of our study was to look at the hospitalizations due to all cancers in the Italian provinces, with specific focus on youngest age groups, namely pediatric population and adults ≤50 years old, which are not covered by screening campaigns. The aim was to provide some data about the hospital admissions of cancer patients including those areas where cancer registries (CRs) have not been activated or provide only a partial population coverage. Actually, more than 50% of the Italian population is not covered by CRs and therefore no data are available on cancer incidence rates concerning all the regions, especially for Southern Italy.

Since the potential and limitations of CRs and HDR have already been addressed [8,9,10,11,12,13,14,15,16], this work might provide a first rough general picture about the burden of cancer in Italy concerning younger people, based on real official national data such as hospitalization records. We were not still able to split the database for many age groups (the analyses by smaller age groups will be presented in future publications). The decision to focus on pediatric population (0–19 years old) and young adults (20–49 years old) is closely related to the need of providing information about the possible impact of environmental threats to human health coming from the carcinogen substances officially classified by IARC (International Agency for Cancer Research, Lyon, France) [19]. Among those, special attention should be paid to fine and coarse particulate pollution, whose cardiovascular, respiratory and cancer effects on human health arise even after exposure below the legal thresholds and have been investigated in many big cities across Europe, including Rome [20,21,22]. However, it was not the aim of this study to specifically look at causal relations between tumors and environmental or personal exposures. 

Recent reports of a significant increase in childhood cancer in Europe [2] and especially Italy [23] has caused concern, pushing some authors to critically reconsider this dominant model of carcinogenesis [24] and to reconsider quality, comparability and methods of analysis of data on childhood cancer [25]. Cancer has become the leading cause of death among children over the first year of age. Even after adjusting for population growth and improved detection of certain types of cancer, we have observed an increased in childhood cancer over the past 40 years. A large increase in cancer cases has been specifically documented in the first year of age, suggesting that the cancers may originate from maternal and fetal exposure to pro-carcinogenic agents, or have an epigenetic or gametic origin [2,26]. These data suggest that exposure to carcinogens from pollution could play a bigger role in causing children cancer than that played by unhealthy personal habits in adults (i.e., cigarette smoking)”. 

Looking only at “rates” might be restrictive when assessing the global burden of cancer diseases and the perception by population. When examining cancer rates in the Campania Region, a big city such as Naples with about 300 new cases per year could show a SHR similar to those of smaller towns with 10–20 new cases per year. For example, the SHR in the large city of Naples was 8.89 in year 2011, which is smaller than the SHR of the smaller town of Isernia with an SHR of 10.43. However, on an absolute scale, more than 300 cases of pediatric cancer would arise in Naples in a single year, which is much higher than 8 pediatric cases per year hospitalized in Isernia. This perceived difference in the number of cancer cases in the population of Naples could contribute to public alarm and panic. Similarly, the overall 280 hospitalizations due to pediatric cancer in Milan (SHR 2011: 5.37) are not considered as a particular problem compared to the 20 cases of Teramo (SHR 2011: 5.73) when looking only at “rates”. We should also look at the problem from an ethical point of view: if we consider as “normal” that cities with a higher number of children must have more incident cancer cases, it could be questioned if we can accept in our society that “more children means more tumors”.

It is interesting to point out that public perception acts in a completely different way (no social alarm) in other areas presenting even more relevant number of pediatric hospitalizations, such as Rome and surroundings (more than 300 new cases per year on average only in the capital city). This would probably suggests that information provided to the population (i.e., the discovery of a huge number of illegal deposits or dumping sites, incineration of dangerous wastes) contribute to create panic and social alarm. Beyond the social consequences of having more children or adults suffering from cancer, we believe it is important for decision makers to have information about the absolute number of cancer cases in order to provide adequate both in-the hospital and in-home-based healthcare facilities to take care of a huge number of patients. 

In this paper, we have presented our data per province and for all cancer types. Of course, we are aware that the highest observed hospitalization rates could reflect problems that are specifically attributable to the biggest cities or to single towns within the province (with the rest of the area not being responsible for the increased rates). In addition, particularly high values of SHR observed in smaller provinces might be the consequence of the higher incidence of specific tumors related to particular (environmental or professional) exposures typical of that territory. Of course, only local CRs are able to accomplish this level of characterization. This is the case of Taranto, where the local cancer registry has recorded higher incidence rates of pediatric tumors than those found out in our analyses [27]. Particular attention should be paid to the higher incidence of cancer in adults (mostly women) in the entire Sardinia Island, where the activity of CRs is still at the initial stages. 

We believe that cancer hospitalization data per province presented in this study may provide a first interesting rough picture of the global problem, that can be further and deeper investigated through the use of local CRs (where and when available). We are also aware that the use of hospitalization records for epidemiological purposes present a series of limitations mainly consisting in the unavoidable “false negative cases”: cancer patients who are not hospitalized because treated at ambulatorial level and consequently not included in the national hospitalizations database. Despite hospitalization records have not been conceived as a primary epidemiological instrument but as an administrative tool, they are completed only after histological exam has allowed a final diagnosis. Therefore, the use of hospitalization records for epidemiological purposes is particularly valuable. Of course, there is the possibility of an under-estimation produced in our study by the presence of cancer patient not hospitalized, which can be detected only by CRs as gold standard methodology. However, the aim of this study was to provide a first picture of the phenomenon at national level, including also those areas not covered by CRs, which could provide detailed data once activated. 

It is interesting to point out that our findings about the incidence of all pediatric cancer in Italy (with about 11,800 new cases per year over a 5-year period) are consistent with AIRTUM projections for years 2016–2020 which estimate about 11,000 new cases of cancer in the age group 0–19 in the National Association of Cancer Registries 2013 Report [28]. 

Data presented in this paper might represent an initial step which should encourage scientists and public bodies to assess the causes of the increasing cancer hospitalization rates in different Italian areas, with the ending of the traditional gap between Northern and Southern Italy in the field of cancer diseases. The role played by environmental pollutants [29], food and water contamination (i.e., pesticides use, toxic wastes, heavy metals, dioxins and others), nutritional, professional and other personal habits should be investigated. Finally, the higher SHR rates displayed in our study in young women (compared to men) aged 20–49 years old should be considered as a very interesting finding, as it is only partially likely to be the effect of population screening campaigns. Actually, mammographic screening campaigns involve older females aged >50 years old and only tests for the prevention of uterine cervix cancer are performed at younger age. Therefore, this latter finding could probably reflect a higher incidence of some specific female tumors (i.e., breast and thyroid cancer) as suggested by AIRTUM Reports in the younger age groups [28,30,31]. 

## 5. Conclusions

Despite the limitations due to the possible underestimation of cancer incidence, it is feasible and potentially useful to use hospitalization records as secondary data source where cancer registries do not cover an entire province or region, in order to provide preliminary information of cancer burden. As expected, the biggest Italian cities showed the highest number of hospitalizations, with well-defined areas being characterized by more pronounced SHR. In addition to the SHR, the absolute number of new cancer cases represents a crucial information for a global assessment of the problem (including healthcare, social, environmental and other evaluations) as well as for adequate planning of healthcare services by decision makers at regional level. Our results over a 5-year period are consistent with AIRTUM projections for years 2016–2020 and call for specific risk assessment programs at local level to search for causal relations with environmental and personal or professionals exposures, that should performed on cancer registries data and case-control studies as the most qualified tool for that. 

## Figures and Tables

**Table 1:** Overall cancer standardized hospitalization rate per region in population aged 0–100 years old (Average Annual Value).

Region	Average Annual SHR
Piemonte	68.84
Valle d’Aosta	87.30
Lombardia	76.64
Trentino Alto Adige	73.72
Veneto	75.19
Friuli Venezia Giulia	86.86
Liguria	84.34
Emilia/Romagna	84.00
Toscana	72.37
Umbria	76.10
Marche	76.22
Lazio	79.92
Abruzzo	67.50
Molise	74.22
Campania	77.46
Puglia	74.93
Basilicata	71.54
Calabria	65.73
Sicilia	73.71
Sardegna	80.51

**Table 2:** Standardized hospitalization rate (SHR) per 100,000 and overall number of new hospitalizations due to cancer per region and province from 2007 to 2011. Pediatric patients aged 0–19 years old.

Region	Province	2007		2008		2009		2010		2011		Mean Values 2007–2011	
		*n*	SHR	*n*	SHR	*n*	SHR	*n*	SHR	*n*	SHR	*n*	SHR
**Northern Italy**													
Lombardia	Como	38	6.50	36	6.15	32	5.47	35	5.98	45	7.69	37	6.36
	Varese	34	3.98	54	6.32	47	5.50	55	6.43	59	6.90	50	5.83
	Cremona	15	4.44	15	4.44	13	3.85	17	5.04	20	5.93	16	4.74
	Mantova	23	5.86	16	4.08	13	3.31	26	6.62	23	5.86	20	5.15
	Brescia	82	6.22	65	4.93	60	4.55	91	6.90	76	5.77	75	5.67
	Pavia	27	5.69	28	5.90	22	4.64	37	7.80	26	5.48	28	5.90
	Bergamo	36	3.06	54	4.58	43	3.65	57	4.84	64	5.43	51	4.31
	**Milano**	**222**	**7.64**	**215**	**7.40**	**229**	**7.88**	**239**	**8.23**	**156**	**5.37**	**212**	**7.30**
	Lodi	10	4.46	14	6.24	14	6.24	18	8.02	12	5.35	14	6.06
	Monza-Brianza									44	5.18	44	5.18
	Sondrio	6	3.33	7	3.89	15	8.33	11	6.11	9	5.00	10	5.33
	Lecco	22	6.42	19	5.55	11	3.21	30	8.76	17	4.96	20	5.78
	**TOTAL**	**515**	**5.86**	**523**	**5.95**	**499**	**5.67**	**616**	**7.01**	**551**	**6.27**	**541**	**6.15**
Veneto	Rovigo	19	9.60	13	6.57	6	3.03	10	5.05	15	7.58	13	6.37
	**Venezia**	**56**	**7.15**	**48**	**6.13**	**45**	**5.75**	**39**	**4.98**	**58**	**7.41**	**49**	**6.28**
	Vicenza	69	7.48	54	5.85	61	6.61	59	6.39	66	7.15	62	6.70
	Treviso	63	6.81	63	6.81	55	5.94	70	7.56	58	6.27	62	6.68
	Verona	39	4.23	44	4.77	60	6.51	50	5.42	54	5.86	49	5.36
	Padova	52	5.70	47	5.15	56	6.14	66	7.24	51	5.59	54	5.96
	Belluno	8	4.21	12	6.31	3	1.58	15	7.89	8	4.21	9	4.84
	**TOTAL**	**306**	**6.31**	**281**	**5.79**	**286**	**5.89**	**309**	**6.37**	**310**	**6.39**	**298**	**6.15**
Emilia-Romagna	Parma	23	5.89	27	6.91	40	10.24	41	10.49	35	8.96	33	8.50
	Rimini	12	3.84	20	6.39	16	5.12	26	8.31	22	7.03	19	6.14
	**Bologna**	**49**	**5.62**	**65**	**7.45**	**54**	**6.19**	**62**	**7.11**	**48**	**5.50**	**56**	**6.37**
	Forlì-Cesena	28	7.68	22	6.03	6	1.64	21	5.76	20	5.48	19	5.32
	Ravenna	15	4.37	17	4.95	17	4.95	10	2.91	18	5.24	15	4.48
	Reggio Emilia	29	5.34	40	7.37	37	6.81	44	8.10	27	4.97	35	6.52
	Piacenza	17	6.64	12	4.69	26	10.16	24	9.38	12	4.69	18	7.11
	Modena	28	4.10	44	6.45	33	4.84	40	5.86	30	4.40	35	5.13
	Ferrara	24	8.81	19	6.97	17	6.24	13	4.77	7	2.57	16	5.87
	**TOTAL**	**225**	**5.57**	**266**	**6.59**	**246**	**6.09**	**281**	**6.96**	**219**	**5.42**	**247**	**6.13**
Piemonte	**Torino**	**108**	**5.22**	**124**	**5.99**	**130**	**6.28**	**143**	**6.91**	**131**	**6.33**	**127**	**6.15**
	Alessandria	16	4.60	26	7.47	41	11.78	30	8.62	20	5.75	27	7.64
	Vercelli	7	4.67	17	11.33	17	11.33	18	12.00	8	5.33	13	8.93
	Novara	26	7.55	28	8.13	17	4.93	16	4.64	14	4.06	20	5.86
	Biella	17	11.01	8	5.18	7	4.53	6	3.89	6	3.89	9	5.70
	Asti	10	5.09	11	5.60	13	6.61	20	10.17	7	3.56	12	6.21
	Cuneo	45	7.81	27	4.68	25	4.34	28	4.86	20	3.47	29	5.03
	Verbano-Cusio-Ossola	2	1.44	8	5.76	10	7.20	18	12.97	2	1.44	8	5.76
	**TOTAL**	**231**	**5.81**	**249**	**6.26**	**260**	**6.53**	**279**	**7.01**	**208**	**5.23**	**245**	**6.17**
Liguria	Imperia	10	5.52	15	8.28	15	8.28	26	14.36	20	11.04	17	9.50
	**Genova**	**56**	**7.94**	**41**	**5.81**	**55**	**7.80**	**50**	**7.09**	**45**	**6.38**	**49**	**7.00**
	La Spezia	8	4.48	17	9.52	16	8.96	14	7.84	10	5.60	13	7.28
	Savona	22	9.63	22	9.63	13	5.69	19	8.31	9	3.94	17	7.44
	**TOTAL**	**96**	**7.42**	**95**	**7.34**	**99**	**7.65**	**109**	**8.43**	**84**	**6.49**	**97**	**7.47**
Trentino-Alto Adige	**Trento**	**32**	**5.62**	**22**	**3.87**	**33**	**5.80**	**37**	**6.50**	**42**	**7.38**	**33**	**5.83**
	Bolzano	31	5.27	15	2.55	24	4.08	20	3.40	21	3.57	22	3.77
	**TOTAL**	**63**	**5.44**	**37**	**3.20**	**57**	**4.93**	**57**	**4.92**	**63**	**5.44**	**55**	**4.79**
Friuli Venezia Giulia	Udine	24	5.11	22	4.69	42	8.95	23	4.90	31	6.61	28	6.05
	**Trieste**	**11**	**5.87**	**13**	**6.94**	**13**	**6.94**	**15**	**8.01**	**12**	**6.41**	**13**	**6.83**
	Gorizia	6	4.97	2	1.66	11	9.10	6	4.97	6	4.97	6	5.13
	Pordenone	20	6.63	15	4.97	24	7.95	27	8.95	13	4.31	20	6.56
	**TOTAL**	**61**	**5.65**	**52**	**4.82**	**90**	**8.34**	**71**	**6.58**	**62**	**5.75**	**67**	**6.23**
Valle d’Aosta	**Aosta**	**10**	**8.11**	**12**	**9.73**	**4**	**3.24**	**14**	**11.36**	**10**	**8.11**	**10**	**8.11**
**Total Northern Italy**		**1446**	**5.71**	**1463**	**5.78**	**1451**	**5.73**	**1665**	**6.58**	**1445**	**5.71**	**1494**	**5.90**
Central Italy													
Lazio	**Roma**	**433**	**10.96**	**387**	**9.80**	**408**	**10.33**	**375**	**9.49**	**357**	**9.04**	**392**	**9.92**
	Frosinone	42	8.95	58	12.36	55	11.72	53	11.29	42	8.95	50	10.65
	Rieti	19	13.72	8	5.78	16	11.55	11	7.94	12	8.66	13	9.53
	Latina	82	14.65	74	13.22	52	9.29	51	9.11	47	8.40	61	10.93
	Viterbo	42	14.83	26	9.18	18	6.35	16	5.65	21	7.41	25	8.68
	**TOTAL**	**618**	**11.44**	**553**	**10.24**	**549**	**10.17**	**506**	**9.37**	**479**	**8.87**	**541**	**10.02**
Toscana	Lucca	29	8.44	19	5.53	21	6.11	17	4.95	27	7.85	23	6.58
	Grosseto	19	10.37	9	4.91	7	3.82	12	6.55	14	7.64	12	6.66
	Arezzo	16	5.10	22	7.01	14	4.46	19	6.06	21	6.70	18	5.87
	Pisa	26	6.96	19	5.09	27	7.23	16	4.29	19	5.09	21	5.73
	Siena	18	7.58	17	7.16	18	7.58	6	2.53	12	5.06	14	5.98
	Pistoia	13	5.00	8	3.08	12	4.62	13	5.00	12	4.62	12	4.46
	Livorno	16	5.61	11	3.86	19	6.66	12	4.21	10	3.50	14	4.77
	**Firenze**	**39**	**4.38**	**46**	**5.17**	**41**	**4.61**	**38**	**4.27**	**31**	**3.48**	**39**	**4.38**
	Massa-Carrara	16	9.64	4	2.41	13	7.83	7	4.22	5	3.01	9	5.42
	Prato	9	3.73	15	6.22	9	3.73	6	2.49	3	1.24	8	3.48
	**TOTAL**	**201**	**6.10**	**170**	**5.16**	**181**	**5.50**	**146**	**4.43**	**154**	**4.67**	**170**	**5.17**
Marche	Pesaro-Urbino	27	7.68	21	5.97	24	6.83	25	7.11	30	8.53	25	7.22
	Ascoli Piceno	32	16.39	21	10.76	31	15.88	17	8.71	16	8.20	23	11.99
	**Ancona**	**31**	**6.89**	**35**	**7.78**	**38**	**8.45**	**28**	**6.22**	**32**	**7.11**	**33**	**7.29**
	Macerata	17	5.61	22	7.26	25	8.25	17	5.61	19	6.27	20	6.60
	Fermo							11	6.72	10	6.11	11	6.42
	**TOTAL**	**107**	**8.23**	**99**	**7.62**	**118**	**9.08**	**98**	**7.54**	**107**	**8.23**	**106**	**8.14**
Abruzzo	Chieti	30	8.32	43	11.93	27	7.49	29	8.05	24	6.66	31	8.49
	L’Aquila	31	11.62	23	8.62	30	11.25	16	6.00	17	6.37	23	8.77
	**Pescara**	**18**	**5.83**	**25**	**8.10**	**24**	**7.77**	**25**	**8.10**	**19**	**6.15**	**22**	**7.19**
	Teramo	40	13.48	21	7.08	32	10.79	25	8.43	17	5.73	27	9.10
	**TOTAL**	**119**	**9.65**	**112**	**9.09**	**113**	**9.17**	**95**	**7.71**	**77**	**6.25**	**103**	**8.37**
Umbria	Terni	27	13.68	20	10.13	13	6.59	19	9.63	16	8.11	19	9.63
	**Perugia**	**46**	**7.47**	**43**	**6.98**	**57**	**9.25**	**27**	**4.38**	**35**	**5.68**	**42**	**6.75**
	**TOTAL**	**73**	**8.98**	**63**	**7.74**	**70**	**8.60**	**46**	**5.65**	**51**	**6.27**	**61**	**7.45**
Molise	Isernia	14	18.25	7	9.12	10	13.03	11	14.34	8	10.43	10	13.03
	**Campobasso**	**17**	**7.94**	**20**	**9.34**	**32**	**14.94**	**34**	**15.87**	**18**	**8.40**	**24**	**11.30**
	**TOTAL**	**31**	**10.66**	**27**	**9.28**	**42**	**14.44**	**45**	**15.47**	**26**	**8.94**	**34**	**11.76**
**Total Central Italy**		**1149**	**9.32**	**1024**	**8.31**	**1073**	**8.70**	**936**	**7.59**	**894**	**7.25**	**1015**	**8.23**
**Southern Italy**													
Campania	Salerno	143	12.16	146	12.42	109	9.27	130	11.06	120	10.21	130	11.02
	**Napoli**	**447**	**11.75**	**449**	**11.81**	**333**	**8.76**	**324**	**8.52**	**338**	**8.89**	**378**	**9.95**
	Caserta	132	11.96	140	12.68	101	9.15	99	8.97	92	8.33	113	10.22
	Avellino	45	10.27	52	11.87	45	10.27	59	13.46	35	7.99	47	10.77
	Benevento	30	10.37	36	12.44	19	6.57	24	8.30	18	6.22	25	8.78
	**TOTAL**	**797**	**11.70**	**823**	**12.09**	**607**	**8.92**	**636**	**9.34**	**603**	**8.86**	**693**	**10.18**
Sicilia	Caltanissetta	32	10.12	37	11.70	36	11.39	30	9.49	33	10.44	34	10.63
	Messina	88	13.82	68	10.68	48	7.54	63	9.90	46	7.23	63	9.83
	Agrigento	46	9.28	28	5.65	34	6.86	38	7.67	35	7.06	36	7.30
	Catania	119	9.65	130	10.55	102	8.28	118	9.57	85	6.90	111	8.99
	**Palermo**	**119**	**8.50**	**102**	**7.29**	**127**	**9.08**	**79**	**5.65**	**95**	**6.79**	**104**	**7.46**
	Siracusa	26	6.16	46	10.89	26	6.16	16	3.79	27	6.39	28	6.68
	Trapani	33	7.22	34	7.43	52	11.37	38	8.31	25	5.47	36	7.96
	Enna	21	11.27	14	7.51	12	6.44	18	9.66	10	5.37	15	8.05
	Ragusa	36	10.49	23	6.70	23	6.70	22	6.41	14	4.08	24	6.88
	**TOTAL**	**520**	**9.47**	**482**	**8.78**	**460**	**8.38**	**422**	**7.69**	**370**	**6.74**	**451**	**8.21**
Puglia	Brindisi	37	8.98	24	5.83	31	7.52	32	7.77	40	9.71	33	7.96
	**Bari**	**150**	**11.26**	**111**	**8.33**	**118**	**8.86**	**110**	**8.26**	**101**	**7.58**	**118**	**8.86**
	Foggia	67	9.45	70	9.88	58	8.18	61	8.61	53	7.48	62	8.72
	Barletta-Andria-Trani							55	11.84	34	7.32	45	9.58
	Lecce	54	6.68	52	6.43	56	6.93	49	6.06	59	7.30	54	6.68
	Taranto	51	8.22	41	6.61	45	7.26	34	5.48	33	5.32	41	6.58
	**TOTAL**	**359**	**9.25**	**298**	**7.68**	**308**	**7.94**	**341**	**8.79**	**320**	**8.24**	**325**	**8.38**
Calabria	Cosenza	56	7.92	47	6.65	56	7.92	53	7.50	56	7.92	54	7.58
	Reggio Calabria	54	8.99	70	11.65	45	7.49	54	8.99	46	7.66	54	8.96
	**Catanzaro**	**44**	**11.93**	**35**	**9.49**	**21**	**5.69**	**50**	**13.56**	**27**	**7.32**	**35**	**9.60**
	Vibo Valentia	15	8.43	9	5.06	12	6.74	18	10.11	10	5.62	13	7.19
	Crotone	21	10.46	13	6.48	10	4.98	18	8.97	9	4.48	14	7.07
	**TOTAL**	**190**	**9.24**	**174**	**8.47**	**144**	**7.00**	**193**	**9.39**	**148**	**7.20**	**170**	**8.26**
Sardegna	Oristano	11	7.95	7	5.06	17	12.29	13	9.39	13	9.39	12	8.82
	Medio Campidano	8	9.20	13	14.94	5	5.75	9	10.35	8	9.20	9	9.89
	**Cagliari**	**40**	**8.17**	**25**	**5.11**	**40**	**8.17**	**33**	**6.74**	**44**	**8.99**	**36**	**7.44**
	Nuoro	14	9.21	9	5.92	10	6.58	11	7.24	13	8.55	11	7.50
	Ogliastra	4	7.45	6	11.18	5	9.31	3	5.59	4	7.45	4	8.20
	Olbia-Tempio	9	6.25	16	11.11	14	9.72	6	4.17	10	6.95	11	7.64
	Sassari	27	9.13	37	12.51	23	7.78	17	5.75	12	4.06	23	7.85
	Carbonia-Iglesias	5	4.84	10	9.68	6	5.81	9	8.71	4	3.87	7	6.58
	**TOTAL**	**118**	**8.06**	**123**	**8.41**	**120**	**8.20**	**101**	**6.90**	**108**	**7.38**	**114**	**7.79**
Basilicata	**Potenza**	**27**	**7.35**	**23**	**6.26**	**25**	**6.81**	**29**	**7.90**	**37**	**10.08**	**28**	**7.68**
	Matera	20	9.78	12	5.87	10	4.89	10	4.89	11	5.38	13	6.16
	**TOTAL**	**47**	**8.22**	**35**	**6.12**	**35**	**6.12**	**39**	**6.82**	**48**	**8.40**	**41**	**7.14**
**Total Southern Italy**		**2031**	**10.02**	**1935**	**9.55**	**1674**	**8.26**	**1732**	**8.55**	**1597**	**7.88**	**1794**	**8.85**

Note: Regional capitals are highlighted in bold.

**Table 3:** Standardized hospitalization rate (SHR) per 100,000 and overall number of new hospitalizations due to cancer per region and province from 2007 to 2011. Adults aged 20–49 years old.

Region	Province	2007		2008		2009		2010		2011		Mean Values 2007–2011	
		*n*	SHR	*n*	SHR	*n*	SHR	*n*	SHR	*n*	SHR	*n*	SHR
**Northern Italy**													
Lombardia	Mantova	291	71.55	288	70.81	276	67.86	291	71.55	324	79.66	294	72.29
	Pavia	359	67.93	380	71.90	341	64.52	331	62.63	378	71.52	358	67.70
	Brescia	894	70.23	918	72.12	901	70.78	939	73.77	876	68.82	906	71.14
	Lecco	211	63.42	224	67.32	225	67.62	200	60.11	219	65.82	216	64.86
	**Milano**	**2694**	**89.19**	**2711**	**89.76**	**2663**	**88.17**	**2434**	**80.59**	**1951**	**64.59**	**2491**	**82.46**
	Como	370	62.42	414	69.84	417	70.35	356	60.06	369	62.25	385	64.98
	Sondrio	120	67.52	138	77.64	135	75.96	126	70.89	109	61.33	126	70.67
	Cremona	240	68.28	231	65.72	215	61.16	246	69.98	215	61.16	229	65.26
	Varese	600	68.94	640	73.53	541	62.16	555	63.77	526	60.43	572	65.77
	Monza-Brianza									514	59.90	514	59.90
	Bergamo	614	54.79	605	53.98	682	60.86	663	59.16	662	59.07	645	57.57
	Lodi	173	74.52	165	71.07	164	70.64	149	64.18	136	58.58	157	67.80
	**TOTAL**	**6566**	**73.72**	**6714**	**75.38**	**6560**	**73.66**	**6290**	**70.62**	**6279**	**64.30**	**6482**	**71.54**
Veneto	Rovigo	139	59.22	165	70.30	186	79.25	210	89.48	185	78.82	177	75.41
	**Venezia**	**588**	**71.07**	**571**	**69.02**	**587**	**70.95**	**577**	**69.75**	**565**	**68.29**	**578**	**69.82**
	Verona	601	65.53	627	68.37	658	71.75	595	64.88	602	65.64	617	67.23
	Padova	648	69.01	673	71.67	651	69.32	593	63.15	579	61.66	629	66.96
	Treviso	548	61.43	577	64.68	576	64.56	519	58.18	531	59.52	550	61.67
	Belluno	145	73.71	122	62.02	119	60.49	102	51.85	114	57.95	120	61.20
	Vicenza	474	54.12	517	59.03	563	64.28	543	62.00	474	54.12	514	58.71
	**TOTAL**	**3143**	**64.37**	**3252**	**66.60**	**3340**	**68.40**	**3139**	**64.29**	**3050**	**62.46**	**3185**	**65.22**
Emilia-Romagna	Rimini	203	62.64	199	61.41	244	75.30	300	92.58	306	94.43	250	77.27
	Ravenna	294	79.53	312	84.40	321	86.83	327	88.46	312	84.40	313	84.72
	Parma	331	77.79	347	81.55	335	78.73	341	80.14	341	80.14	339	79.67
	**Bologna**	**692**	**72.99**	**762**	**80.37**	**739**	**77.94**	**742**	**78.26**	**742**	**78.26**	**735**	**77.56**
	Modena	496	72.61	506	74.08	506	74.08	452	66.17	533	78.03	499	72.99
	Ferrara	265	80.76	262	79.85	282	85.94	290	88.38	256	78.02	271	82.59
	Piacenza	223	81.59	206	75.37	218	79.76	195	71.34	211	77.20	211	77.05
	Forlì-Cesena	253	65.18	284	73.17	255	65.70	285	73.43	297	76.52	275	70.80
	Reggio Emilia	392	74.45	399	75.78	392	74.45	408	77.49	380	72.17	394	74.87
	**TOTAL**	**3149**	**73.81**	**3277**	**76.81**	**3292**	**77.16**	**3340**	**78.28**	**3378**	**79.17**	**3287**	**77.05**
Piemonte	Alessandria	316	80.79	337	86.16	315	80.53	306	78.23	297	75.93	314	80.33
	Vercelli	111	67.41	118	71.67	108	65.59	107	64.98	121	73.49	113	68.63
	Biella	111	67.35	114	69.17	123	74.63	143	86.76	121	73.42	122	74.27
	Novara	249	68.65	248	68.38	289	79.68	257	70.86	262	72.24	261	71.96
	Verbano-Cusio-Ossola	117	77.38	108	71.43	109	72.09	100	66.14	100	66.14	107	70.64
	**Torino**	**1346**	**62.31**	**1357**	**62.82**	**1383**	**64.03**	**1396**	**64.63**	**1396**	**64.63**	**1376**	**63.68**
	Cuneo	307	54.25	309	54.60	342	60.43	332	58.67	341	60.26	326	57.64
	Asti	135	66.66	128	63.20	127	62.71	111	54.81	119	58.76	124	61.23
	**TOTAL**	**2692**	**64.66**	**2719**	**65.31**	**2796**	**67.16**	**2752**	**66.11**	**2757**	**66.23**	**2743**	**65.89**
Liguria	La Spezia	175	86.82	180	89.30	160	79.38	153	75.90	165	81.86	167	82.65
	**Genova**	**599**	**78.90**	**679**	**89.44**	**649**	**85.49**	**545**	**71.79**	**596**	**78.51**	**614**	**80.83**
	Savona	194	77.39	193	76.99	207	82.57	179	71.40	187	74.59	192	76.59
	Imperia	147	76.21	185	95.91	150	77.77	139	72.06	131	67.92	150	77.97
	**TOTAL**	**1115**	**79.40**	**1237**	**88.09**	**1166**	**83.03**	**1016**	**72.35**	**1079**	**76.84**	**1123**	**79.94**
Friuli Venezia Giulia	Udine	381	74.10	419	81.49	404	78.57	376	73.13	397	77.21	395	76.90
	Pordenone	232	74.00	221	70.49	193	61.56	203	64.75	238	75.91	217	69.34
	**Trieste**	**169**	**81.64**	**161**	**77.77**	**159**	**76.81**	**136**	**65.70**	**148**	**71.49**	**155**	**74.68**
	Gorizia	116	88.62	111	84.80	106	80.98	103	78.69	83	63.41	104	79.30
	**TOTAL**	**898**	**77.04**	**912**	**78.24**	**862**	**73.95**	**818**	**70.18**	**866**	**74.29**	**871**	**74.74**
Trentino-Alto Adige	**Trento**	**334**	**64.51**	**319**	**61.61**	**366**	**70.69**	**320**	**61.80**	**373**	**72.04**	**342**	**66.13**
	Bolzano	247	47.60	245	47.22	260	50.11	226	43.55	269	51.84	249	48.06
	**TOTAL**	**581**	**56.05**	**564**	**54.41**	**626**	**60.39**	**546**	**52.66**	**642**	**61.93**	**592**	**57.09**
Valle d’Aosta	**Aosta**	**88**	**70.63**	**92**	**73.84**	**78**	**62.60**	**82**	**65.81**	**73**	**58.59**	**83**	**66.29**
**Total Northern Italy**		**18,232**	**70.26**	**18,767**	**72.32**	**18,720**	**72.14**	**17,983**	**69.30**	**18,124**	**67.61**	**14,625**	**70.33**
**Central Italy**													
Lazio	**Roma**	**3555**	**87.10**	**3796**	**93.00**	**3804**	**93.20**	**3576**	**87.61**	**3436**	**84.18**	**3633**	**89.02**
	Latina	493	86.45	473	82.95	433	75.93	481	84.35	449	78.74	466	81.68
	Frosinone	417	83.53	406	81.32	421	84.33	416	83.33	378	75.72	408	81.65
	Rieti	139	92.91	120	80.21	119	79.54	132	88.23	105	70.18	123	82.21
	Viterbo	230	73.93	217	69.75	251	80.68	267	85.83	191	61.40	231	74.32
	**TOTAL**	**4834**	**86.14**	**5012**	**89.31**	**5028**	**89.60**	**4872**	**86.82**	**4559**	**81.24**	**4861**	**86.62**
Toscana	Livorno	266	83.89	268	84.52	271	85.47	252	79.48	229	72.22	257	81.12
	Lucca	269	72.02	326	87.29	297	79.52	289	77.38	264	70.68	289	77.38
	Massa-Carrara	142	74.87	175	92.27	167	88.06	145	76.46	130	68.55	152	80.04
	Pisa	295	72.63	317	78.04	311	76.56	274	67.46	276	67.95	295	72.53
	**Firenze**	**627**	**68.02**	**651**	**70.62**	**648**	**70.30**	**651**	**70.62**	**596**	**64.66**	**635**	**68.84**
	Siena	177	69.87	182	71.84	145	57.24	165	65.13	161	63.55	166	65.53
	Pistoia	187	66.85	192	68.64	212	75.79	173	61.84	177	63.27	188	67.28
	Prato	166	66.79	137	55.12	134	53.91	167	67.19	157	63.16	152	61.23
	Arezzo	246	73.49	224	66.92	194	57.96	214	63.93	204	60.95	216	64.65
	Grosseto	162	79.47	161	78.98	160	78.49	134	65.73	124	60.83	148	72.70
	**TOTAL**	**257**	**71.90**	**2633**	**74.62**	**2539**	**71.96**	**2464**	**69.83**	**2318**	**65.70**	**2042**	**70.80**
Marche	**Ancona**	**350**	**76.05**	**399**	**86.70**	**365**	**79.31**	**369**	**80.18**	**392**	**85.18**	**375**	**81.48**
	Pesaro-Urbino	299	82.85	281	77.86	293	81.19	275	76.20	289	80.08	287	79.64
	Fermo							127	75.49	125	74.31	126	74.90
	Ascoli Piceno	302	147.68	296	144.75	324	158.44	139	67.97	144	70.42	241	117.85
	Macerata	256	82.84	217	70.22	274	88.66	226	73.13	217	70.22	238	77.01
	**TOTAL**	**1207**	**90.44**	**1193**	**89.39**	**1256**	**94.11**	**1136**	**75.59**	**1167**	**77.65**	**1192**	**85.44**
Abruzzo	L’Aquila	250	84.50	288	97.35	226	76.39	215	72.67	209	70.64	238	80.31
	**Pescara**	**212**	**67.48**	**221**	**70.34**	**214**	**68.11**	**210**	**66.84**	**217**	**69.07**	**215**	**68.37**
	Teramo	225	72.41	208	66.94	219	70.48	216	69.51	213	68.55	216	69.58
	Chieti	254	66.72	261	68.56	253	66.46	238	62.52	238	62.52	249	65.36
	**TOTAL**	**941**	**72.30**	**978**	**75.15**	**912**	**70.08**	**879**	**67.54**	**877**	**67.39**	**917**	**70.49**
Umbria	Terni	143	66.97	188	88.05	194	90.86	181	84.77	172	80.55	176	82.24
	**Perugia**	**419**	**65.76**	**484**	**75.96**	**492**	**77.21**	**521**	**81.77**	**478**	**75.02**	**479**	**75.14**
	**TOTAL**	**562**	**66.06**	**672**	**78.99**	**686**	**80.64**	**702**	**82.52**	**650**	**76.41**	**654**	**76.92**
Molise	**Campobasso**	**189**	**85.28**	**200**	**90.24**	**187**	**84.38**	**190**	**85.73**	**165**	**74.45**	**186**	**84.02**
	Isernia	66	76.81	53	61.68	65	75.64	63	73.31	60	69.82	61	71.45
	**TOTAL**	**255**	**82.91**	**253**	**82.26**	**252**	**81.94**	**253**	**82.26**	**225**	**73.16**	**248**	**80.51**
**Total Central Italy**		**10,336**	**79.91**	**10,741**	**83.04**	**10,673**	**82.52**	**10,306**	**78.65**	**9796**	**74.76**	**10,370**	**79.78**
**Southern Italy**													
Campania	**Napoli**	**2482**	**77.47**	**2353**	**73.45**	**2416**	**75.41**	**2358**	**73.60**	**2382**	**74.35**	**2398**	**74.86**
	Caserta	659	67.93	680	70.09	699	72.05	650	67.00	679	69.99	673	69.41
	Salerno	730	64.81	804	71.38	795	70.58	804	71.38	773	68.63	781	69.36
	Avellino	313	71.48	309	70.57	320	73.08	319	72.85	277	63.26	308	70.25
	Benevento	193	67.73	210	73.69	211	74.04	183	64.22	153	53.69	190	66.67
	**TOTAL**	**4377**	**72.67**	**4356**	**72.32**	**4441**	**73.73**	**4314**	**71.62**	**4264**	**70.79**	**4350**	**72.23**
Sicilia	Messina	557	85.84	527	81.21	512	78.90	491	75.66	535	82.44	524	80.81
	Caltanissetta	206	75.25	184	67.21	199	72.69	218	79.63	219	80.00	205	74.96
	Enna	137	80.20	123	72.01	161	94.25	126	73.76	136	79.62	137	79.97
	Catania	1011	91.17	918	82.78	951	85.76	874	78.81	864	77.91	924	83.29
	Siracusa	288	69.90	307	74.51	317	76.93	304	73.78	288	69.90	301	73.00
	Agrigento	312	69.97	270	60.55	266	59.65	306	68.62	299	67.05	291	65.17
	**Palermo**	**854**	**67.62**	**835**	**66.11**	**824**	**65.24**	**799**	**63.26**	**803**	**63.58**	**823**	**65.16**
	Trapani	265	62.52	299	70.54	264	62.28	238	56.15	244	57.56	262	61.81
	Ragusa	201	63.70	178	56.41	248	78.59	208	65.91	171	54.19	201	63.76
	**TOTAL**	**3831**	**75.67**	**3641**	**71.92**	**3742**	**73.91**	**3564**	**70.39**	**3559**	**70.30**	**3667**	**72.44**
Puglia	Lecce	648	81.48	660	82.98	650	81.73	644	80.97	625	78.58	645	81.15
	Taranto	465	77.83	431	72.14	427	71.47	424	70.97	457	76.49	441	73.78
	**Bari**	**1180**	**91.83**	**1213**	**94.40**	**1219**	**94.87**	**926**	**72.07**	**977**	**76.04**	**1103**	**85.84**
	Foggia	487	77.00	487	77.00	439	69.41	489	77.32	455	71.94	471	74.53
	Brindisi	300	74.06	300	74.06	298	73.57	290	71.59	281	69.37	294	72.53
	Barletta-Andria-Trani							248	59.88	269	64.95	259	62.42
	**TOTAL**	**3080**	**82.90**	**3091**	**83.20**	**3033**	**81.64**	**3021**	**81.32**	**3064**	**82.47**	**3058**	**67.41**
Calabria	Cosenza	473	64.64	513	70.10	477	65.18	514	70.24	541	73.93	504	68.82
	**Catanzaro**	**255**	**69.53**	**263**	**71.71**	**218**	**59.44**	**253**	**68.98**	**246**	**67.07**	**247**	**67.35**
	Reggio Calabria	391	69.81	424	75.70	372	66.42	364	64.99	369	65.88	384	68.56
	Crotone	128	72.92	123	70.07	95	54.12	113	64.37	115	65.51	115	65.40
	Vibo Valentia	120	72.94	119	72.34	106	64.43	91	55.32	97	58.96	107	64.80
	**TOTAL**	**1367**	**68.40**	**1442**	**72.15**	**1268**	**63.44**	**1335**	**66.79**	**1368**	**68.44**	**1356**	**67.84**
Sardegna	Ogliastra	48	82.63	37	63.69	48	82.63	46	79.19	56	96.40	47	80.91
	Carbonia-Iglesias	141	110.42	122	95.54	112	87.71	136	106.51	107	83.79	124	96.79
	Olbia-Tempio	119	73.83	115	71.35	104	64.53	133	82.52	131	81.28	120	74.70
	Sassari	278	82.78	282	83.97	265	78.91	265	78.91	270	80.40	272	80.99
	**Cagliari**	**543**	**93.15**	**533**	**91.44**	**525**	**90.06**	**540**	**92.64**	**456**	**78.23**	**519**	**89.10**
	Oristano	109	67.53	109	67.53	142	87.98	135	83.64	123	76.20	124	76.58
	Nuoro	167	105.65	159	100.59	133	84.14	122	77.18	119	75.29	140	88.57
	Medio Campidano	77	74.78	85	82.55	100	97.11	87	84.49	61	59.24	82	79.63
	**TOTAL**	**1482**	**87.79**	**1442**	**85.42**	**1429**	**84.65**	**1464**	**86.72**	**1323**	**78.37**	**1428**	**84.59**
Basilicata	**Potenza**	**245**	**64.55**	**249**	**65.60**	**253**	**66.65**	**278**	**73.24**	**281**	**74.03**	**261**	**68.81**
	Matera	156	77.33	134	66.43	148	73.37	140	69.40	121	59.98	140	69.30
	**TOTAL**	**401**	**68.99**	**383**	**65.89**	**401**	**68.98**	**418**	**71.91**	**402**	**69.15**	**401**	**68.98**
**Total Southern Italy**		**14,538**	**76.24**	**14,355**	**75.28**	**14,314**	**75.06**	**14,116**	**74.02**	**13,980**	**73.31**	**11,467**	**74.78**

Regional capitals are highlighted in bold.
